# Genetic Connectivity among and Self-Replenishment within Island Populations of a Restricted Range Subtropical Reef Fish

**DOI:** 10.1371/journal.pone.0049660

**Published:** 2012-11-21

**Authors:** Martin H. van der Meer, Jean-Paul A. Hobbs, Geoffrey P. Jones, Lynne van Herwerden

**Affiliations:** 1 Molecular Ecology and Evolution Laboratory, Australian Tropical Sciences and Innovation Precinct, James Cook University, Townsville, Queensland, Australia; 2 School of Marine and Tropical Biology, James Cook University, Townsville, Queensland, Australia; 3 ARC Centre of Excellence for Coral Reef Studies, James Cook University, Townsville, Queensland, Australia; 4 The Oceans Institute and School of Plant Biology, The University of Western Australia, Crawley, Western Australia, Australia; 5 Australian Institute of Marine Science, Perth, Western Australia, Australia; 6 Centre for Sustainable Tropical Fisheries and Aquaculture, James Cook University, Townsville, Queensland, Australia; Leibniz Center for Tropical Marine Ecology, Germany

## Abstract

Marine protected areas (MPAs) are increasingly being advocated and implemented to protect biodiversity on coral reefs. Networks of appropriately sized and spaced reserves can capture a high proportion of species diversity, with gene flow among reserves presumed to promote long term resilience of populations to spatially variable threats. However, numerically rare small range species distributed among isolated locations appear to be at particular risk of extinction and the likely benefits of MPA networks are uncertain. Here we use mitochondrial and microsatellite data to infer evolutionary and contemporary gene flow among isolated locations as well as levels of self-replenishment within locations of the endemic anemonefish *Amphiprion mccullochi,* restricted to three MPA offshore reefs in subtropical East Australia. We infer high levels of gene flow and genetic diversity among locations over evolutionary time, but limited contemporary gene flow amongst locations and high levels of self-replenishment (68 to 84%) within locations over contemporary time. While long distance dispersal explained the species’ integrity in the past, high levels of self-replenishment suggest locations are predominantly maintained by local replenishment. Should local extinction occur, contemporary rescue effects through large scale connectivity are unlikely. For isolated islands with large numbers of endemic species, and high local replenishment, there is a high premium on local species-specific management actions.

## Introduction

It is widely accepted that life within the world’s oceans, especially within highly diverse coral reefs, is under an increasing threat in the 21^st^ century [1]. New management strategies are being developed in a bid to protect marine life from a range of anthropogenic impacts [2], [3]. One of the most popular approaches has been the establishment of no-take Marine Protected Areas (MPAs) whose efficacy in conserving biodiversity continues to be debated. While appropriately designed MPA networks can encompass a high proportion of species [4] and genetic diversity [5], the degree to which reserves contribute to the long term persistence of locations and maintain natural evolutionary processes is uncertain. A major factor that dictates how well MPAs work, is the extent of larval connectivity among locations [6], including links between protected and unprotected areas and among different nodes in MPA networks [7], [8].

Historically, the pelagic larval stage of most marine species was thought to result in broad scale larval dispersal aided by ocean currents [9]. This holds true over evolutionary time scales where the occasional long distance dispersal of pelagic larvae acting as agents of gene flow, have connected distant locations [10], maintained high levels of genetic diversity [11], [12] and thereby helped reduce a species risk of extinction [13]. However, a growing number of studies focusing on contemporary time scales show high levels of self-recruitment [7], [8], [14]. Although none of these studies show 100% self-recruitment, and the scales of contemporary connectivity are only just beginning to be assessed [15], this finding suggests that the appropriate scale and distance between MPAs may indeed be smaller than previously assumed [8], [16], [17]. Thus, connectivity operates over two time scales: evolutionary and contemporary. Most traditional population genetic studies infer evolutionary connectivity [18] (gene flow) using mtDNA to capture the longer term signals of dispersal [19]. In recent years, a range of new statistical software (e.g. STRUCTURE [20], DAPC [21], Migrate-n [22], BAYESASS [23]) has become available and is increasingly being applied to population genetic studies [24] to infer contemporary connectivity using msatDNA to capture the shorter term signals of dispersal. Sometimes there is a 'lack of congruence' between connectivity operating over different time scales (evolutionary and contemporary). For example, coral trout (*Plectropomus maculatus)* and stripey snapper (*Lutjanus carponotatus*) lack spatial genetic structure along the GBR (spanning more than 1000 km) on evolutionary time scales using mtDNA [25], while comprehensive parentage analyses of very large sample sizes at much smaller spatial scales using msatDNA, identified high levels of both local- and self-recruitment for both species [8]. The lack of spatial genetic structure in mtDNA sequence data is not uncommon [26], [27] since only a few recruits per generation are sufficient to maintain spatial genetic homogeneity on evolutionary time scales [28], [29]. Together these tools and analyses (mtDNA and msatDNA) are useful because they provide a more holistic picture of connectivity (gene flow) and retention over a range of spatial and temporal scales [5], [30–35].

The evolution of island faunas is interesting because they are clearly punctuated with evolutionary periods of colonization and gene flow, evidenced by the wide distribution of the same species across isolated locations [36], yet they have presumed low levels of contemporary gene flow. Isolated islands are a conservation priority due to their high level of endemism and high rates of extinction [37]. Species endemic to isolated islands have an increased risk of extinction because they often exhibit a number of vulnerable biological (e.g. flightlessness) [36–38], ecological (e.g. small populations, habitat specialists) [39] and genetic traits (e.g. low gene flow and genetic diversity) [40]. Management plans identify endemic species as a conservation priority; however, effective protection of vulnerable species requires estimates of gene flow (evolutionary and contemporary) between isolated locations and estimates of genetic diversity [41]. Likewise, effective management strategies need to conserve both species and genetic diversity in order to maximise ecosystem and population resilience [42]. Conservation of genetic diversity is an IUCN priority [43] as it provides the raw material for the maintenance of species over evolutionary time scales and provides a basis for responses to rapid environmental change and natural selection [42–44], where a reduced genetic diversity has been correlated with decreased fitness [45].

**Figure 1:**
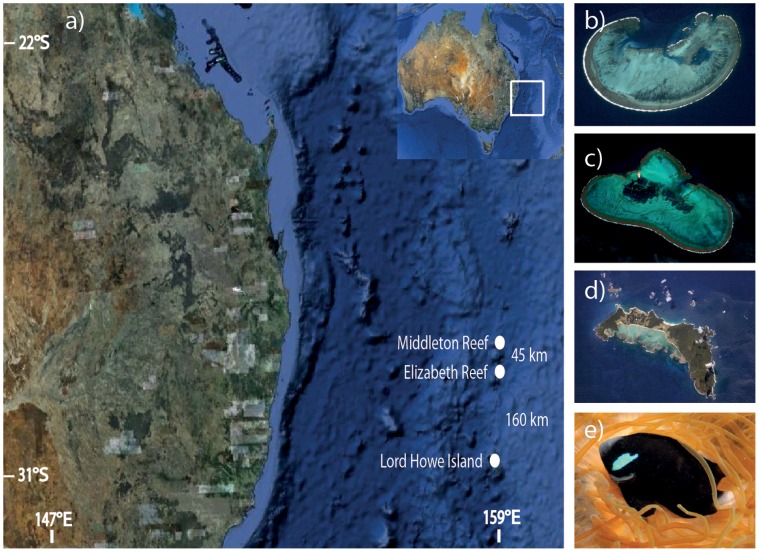
Location maps and focal species. (A) Goole Earth image of eastern Australia showing Middleton Reef (MR), Elizabeth Reef (ER) and Lord Howe Island (LHI) in the Southwest Pacific Ocean, to the southeast of the Great Barrier Reef. Aerial photographs of MR (B); ER (C) and LHI (D), indicating both the outside (LHI) and Lagoon (LHIL) sample sites. (E) *Amphiprion mccullochi* in its host anemone *Entacmaea quadricolor* (Photo courtesy of Justin Gilligan).

In this study we examine evolutionary and contemporary levels of gene flow in the McCulloch’s anemonefish (*Amphiprion mccullochi*), an endemic to three isolated locations in the South-West Pacific Ocean, 600 km off Australia’s east coast (Figure 1a − e). Due to this species being found at only three locations, we were able to sample all known locations leaving no 'ghost' populations un-sampled, giving us a high level of confidence and statistical power in our estimates of gene flow. This species is important as it is potentially at risk of extinction because (i) its geographic range is among the smallest for coral reef fishes (ii) it's an extreme habitat specialist due to its obligate relationship with only one host species of anemone [46] and; (iii) throughout its range it has very low abundance [47–49], except for an extremely small area of habitat at Lord Howe Island Lagoon (LHIL), which supports 92% of the world’s *A. mccullochi* population [48]. Lord Howe Island is a World Heritage Area because it accommodates significant ongoing biological and ecological processes in the development and evolution of coastal, terrestrial, freshwater and marine ecosystems [50]. The island is an endemic hotspot and contains significant habitats for *in-situ* conservation of biological diversity, including threatened species of exceptional conservation value [50]. The efficacy of reserves to reduce extinction risk will depend on evolutionary and contemporary levels of gene low among these isolated locations. We were particularly interested in whether the high abundance of McCulloch’s anemonefish in the lagoon at Lord Howe Island (LHIL) will act to export migrants and help replenish other low abundance locations at greater risk of local extinction.

The aims of this study are fourfold: (i) to determine the patterns and levels of gene flow between locations over evolutionary time scales; (ii) to determine the patterns and levels of gene flow between locations over contemporary time scales; (iii) to infer levels of self-replenishment (as a proxy for self-recruitment) and recent migration (iv) to measure population genetic diversities at all locations as an indicator of potential resilience of populations to environmental change and extinction.

## Materials and Methods

We applied a range of traditional and modern frequency and Bayesian based molecular tools to establish evolutionary and contemporary levels of phylogenetic and population genetic structure. This resulted in a comprehensive understanding of gene flow in this study system and together these molecular tools provided a complete view of different parts of the dispersal kernel [27]. Due to the large number of analyses, we present only methods related to this study below, whilst general Material and Methods such as laboratory techniques and in depth analyses are presented in van der Meer et al. [51], [52]. While this study uses small sample sizes at each location (25−33), typical of population genetic analyses to date [34], [53]; it has the potential to suffer from low statistical power to infer msatDNA genetic differentiation between locations. However, power can be increased either by (i) having more samples, (ii) adding more loci or (iii) adding loci with many alleles [54]. For ethical reasons, taking a larger sample size in a rare endemic species is not sound. Thus, we used many (n = 18) loci that had high allelic richness, to combat the low statistical power of a small sample size and thereby, combined with no un-sampled 'ghost' populations, greatly increase the statistical power to detect msatDNA genetic differentiation between locations.

### Ethics Statement

The main aim of this study was to determine the patterns and levels of gene flow between isolated locations, using the endemic McCulloch’s anemonefish (*Amphiprion mccullochi*) as a model organism. Since this species is rare at two locations (Middleton and Elizabeth Reefs) and all three locations are either World (LHI) or National Heritage (MR, ER) listed, sacrificing individual fish (particularly new recruits) at the ideal scale required for parentage based analyses (hundreds of individuals), is not feasible. Thus a of118 *A. mccullochi* fin clips were taken from four locations, MR (n = 30) [47], ER (n = 25) [47], outside the lagoon at LHI (LHI, n = 33) and within the LHI Lagoon (LHIL, n = 30) [48] using clove oil and hand nets (Permit Numbers: LHIMP08/R01, 003-RRRWN-110211-02, P11/0035-1.0; Animal ethics approval: A1605).

### Study System and Species

*A. mccullochi* inhabits anemones within the coral rich areas of lagoon and seaward reefs at Elizabeth Reef (ER), Middleton Reef (MR) and Lord Howe Island (LHI).The three sites have extensive shallow reefs (<30 m depth) enclosed within MPAs which are separated from each other by deep ocean (>2000 m depth).

### Gene Flow between Locations - Evolutionary Time Scales

#### The mtDNA phylogenetic analysis

The four most commonly used phylogenetic analyses were performed on the aligned mtDNA (D Loop) sequence data as described in [51], [52] and we assigned well supported distinct phylogenetic lineages as management units (MU) [55]. A MU is a population that lacks reciprocal monophyly for mtDNA haplotypes, yet has divergent haplotype frequencies [55], as found here. A Minimum Spanning Tree (MST) was generated to explicitly identify shared haplotypes between *A. mccullochi* from the four locations.

#### Quantifying the level of evolutionary gene flow

Evolutionary migration rates and effective population sizes of *A. mccullochi* were estimated between or within each of the four locations using MIGRATE-n 2.4.3 (http://popgen.sc.fsu.edu/Migrate-n.Html) [22]. Due to the previously identified secondary contact between *A. mccullochi* and *A. akindynos* [51] and since MU were not differentiated geographically, both the Stepping-stone and Island-n migration models were not appropriate as priors for the dataset; rather Migrate-n input files had to be modified and customised. We split the mtDNA data in three ways (i) two groups representing the two admixed lineages: Group 1 (MU 1−2) and Group 2 (MU 3−5) to estimate evolutionary migration between lineages; migration was then compared within Groups (ii) between MU 1 and 2 in Group 1 and; (iii) between MU 3, 4 and 5 in Group 2. We set the datatype to an F84 mutation model and the migration rate parameters for mtDNA (θ and M to a maximum of 0.1 and 1000, respectively) to conduct Bayesian analysis using one long chain that sampled every 100th of 100 k sampled trees and applied a 20 k iteration burn-in. All parameters converged and fell within the 90% CI yielding values for θ and M (mutation-scaled migration rate) per location.

### Gene Flow between Locations - Contemporary Time Scales

#### Patterns of gene flow (msatDNA)

To establish spatial population partitioning in msatDNA, we used three molecular analytical tools: (i) discriminant analysis of principal components (DAPC) [21] was used to discriminate between the four locations, yielding scatterplots of discriminant functions based on the spatial distributions of microsatellite genotypes. DAPC also provided posterior probabilities of population assignments for each individual; (ii) a likelihood-based assignment method was used in GeneClass2 [56]–[58] to determine significant inter-location gene flow and (iii) STRUCTURE V2.3 [20], [59] was used to identify contemporary gene flow between the four locations by applying an Admixture model for 1 M iterations with a 100 k iteration burn-in.

#### Quantifying the level of contemporary gene flow

Contemporary migration rates and effective population sizes of *A. mccullochi* were estimated between each of the four locations using MIGRATE-n 2.4.3 as above. However, we tested a combination of various: migration priors (FST and OWN: isolation-by-distance) and custom-migration models (Stepping-stone, Island-n and variable Theta only); all with a constant mutation rate over all loci. A Log Maximum-Likelihood analysis (Ln ML) comparing all possible combinations selected: migration prior (FST), custom-migration model (migration model with variable Theta) and constant mutation rate over all loci.We set the datatype to Microsatellite (a simple electrophoretic ladder model with stepwise mutation) and the migration rate parameters for msatDNA (θ and M were both set to a maximum of 100) to conduct Bayesian analysis using one long chain that sampled every 100th of 100 k sampled trees and applied a 20 k iteration burn-in. All parameters converged and fell within the 90% CI yielding values for θ and M (mutation-scaled migration rate) for each locus per location.

### Inferred Levels of Self-replenishment and Recent Migration

This study did not sample new anemonefish recruits in order to determine self-recruitment as in [8]. However, we used BAYESASS v3 [23], a program specifically designed for population genetic studies that estimates recent migration rates (past 2−3 generations) between populations (or locations); conversely, this program also has the ability to estimate any individuals not migrating (i.e. self-replenishing). BAYESASS accurately estimates migration rates when the assumptions of the inference model are not violated and genetic differentiation is not too low (F_st_≥0.05); however, when the assumptions are violated, accurate estimates are obtained only when migration rates are very low (*m* = 0.01) and genetic differentiation is high (F_st_≥0.10) [60]. We used BAYESASS v3 to estimate both self-replenishment (as a proxy for self-recruitment) and recent migration between locations; with a MCMC chain, consisting of a total of 11 M steps, a 2 M step burn in and a sampling interval of 100 k, with prior values for migration rate, allele frequency and inbreeding coefficient of 0.95, 0.95 and 0.95, respectively. These priors were selected because they gave acceptance rates of between 20 and 40% [60]. Ten separate runs assessed convergence of the MCMC to evaluate consistency of the results obtained from these inferences.

### Population Genetic Diversities

Molecular diversity indices for mtDNA - haplotype diversity (h); nucleotide diversity (*π*) and for msatDNA - genetic diversity (gd), were estimated in ARLEQUIN 3.5 [61]. Haplotype (*h*) and nucleotide diversities (*π*) of the data were interpreted as either low with specified cut-off values of *h* and *π* (%) were <0.5 or high if values of *h* and *π* (%) were >0.5 [62].

## Results

### Summary Statistics

Three hundred and twenty-two base pairs of mtDNA D-loop were resolved for 105 *Amphiprion mccullochi* individuals. There were a total of forty-six polymorphic sites, of which forty were parsimony informative (six singletons). Allelic diversity was lowest at LHI-L and highest at LHI, whilst F_IS_ did not differ significantly across the three regions surveyed (F_IS_ = 0.07, p = 0.97; Table S1). Elizabeth Reef had the most private alleles, 13 across 17 loci, while the remaining three populations had 12 private alleles each across all loci (Table S1). Of the 17 msatDNA loci: (i) significant single-locus departures from HWE were detected in nine of sixty-eight tests at the population level before FDR correction and two afterwards (LHIL: Am1; ER: Am11); similarly, seven single-locus HWE departures were detected at the regional level before FDR and six afterwards (Table S1); (ii) null alleles were identified in ER (Am6, Am7, Am11, Am19), MR (Am11, Am17), LHI (Am4, Am7) and LHI-L (Am11, Am19) and (iii) of 544 locus × locus exact tests for linkage disequilibrium (136 per population), only 17 were significant before FDR and one after FDR correction (Am6) [63]. Loci that were not in HWE and had null alleles (i.e. Am1, Am11, Am14, Am17, Am19) were not used in subsequent analyses (ARLEQUIN, STRUCTURE, and MIGRATE-n). Detailed summary statistics, mtDNA and msatDNA AMOVA between regions, msatDNA AMOVA by loci, pairwise population comparisons and genetic diversity indices are presented in Supporting Information (S1, S2, S3, S4 and S5 respectively).

### Gene Flow between Locations - Evolutionary Time Scales

#### Synopsis

**Figure 2:**
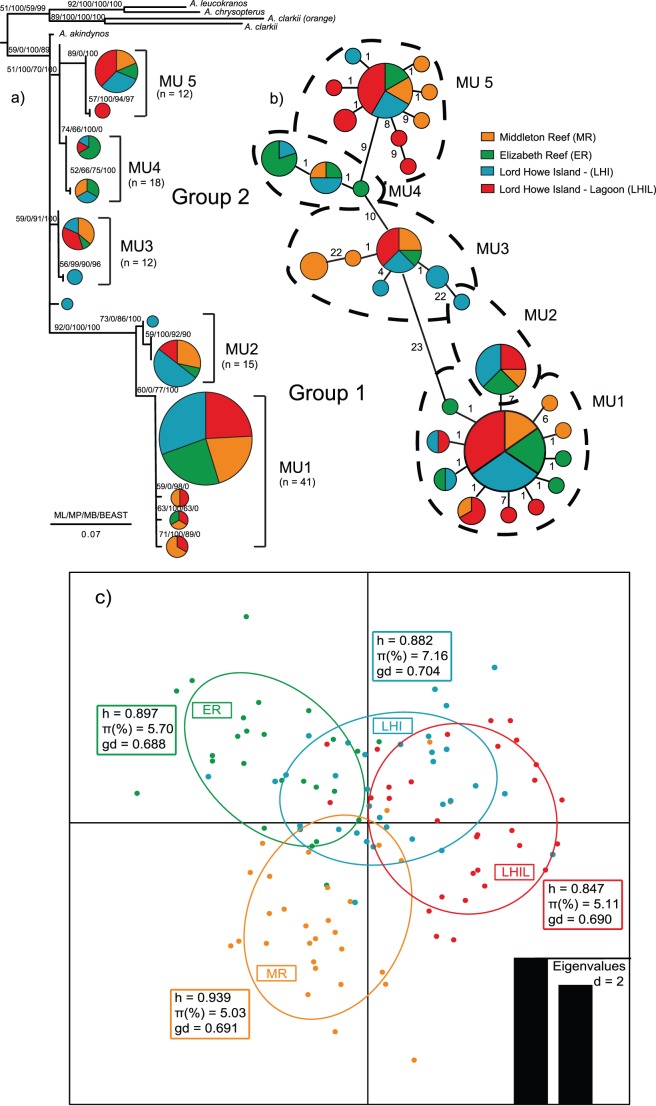
mtDNA and msatDNA gene tic analyses for *Amphiprion. mccullochi*. a) A phylogram of mtDNA (D-Loop) sequences from 118 *A. mccullochi* individuals from Elizabaeth Reef, Middleton Reef and Lord Howe Island. This represents the best ML tree from 10 individual analyses. Numbers on branches indicate support for each clade, based on phylogenetic analyses. b) Haplotype minimum spanning tree (MST) with the number of substitutions between haplotypes indicated on connectors. Different coloured fills represent each of the four populations from the three reefs as shown on the key to the figure. c) Scatterplots of the discriminant analysis of principal components of the microsatellite data for four *Amphiprion mccullochi* populations using geographic sample site as priors for genetic clusters. Individual genotypes appear as dots surrounded by 95% inertia ellipses. Eigenvalues show the amount of genetic information contained in each successive principal component with × and y axes constituting the first two principle components, respectively. Boxes indicate haplotype (*h*), nucleotide (*π*) and genetic diversity (gd) indices for *A. mccullochi*.

*A. mccullochi* mtDNA suggested the existence of two evolutionary lineages (Groups) consisting of a total of five MU with each location being represented in all MU (Figure 2 a, b). High levels of evolutionary gene flow were found between spatially intermixed MU but this was reduced between Groups 1 and 2, which themselves were also spatially intermixed. This suggests that evolutionary gene flow exists between all locations occupied by *A. mccullochi*. The relative percentage of each geographic location within different MU suggests geographic structure and should guide future population monitoring and demographic studies to better inform management.

#### The mtDNA phylogenetic analysis

(Figure 2a) showed two major groups and five distinct management units (MU): MU 1 (n = 41), MU 2 (n = 15), MU 3 (n = 12), MU 4 (n = 18) and MU 5 (n = 12) with a total of 30 haplotypes (Figure 2b). All locations were relatively evenly represented within the two groups: Group 1 (MR = 23, ER = 21, LHI = 32, LHIL = 25) and Group 2 (MR = 21, ER = 21, LHI = 29, LHIL = 29). However, some locations had markedly different proportional representation within some MU (in bold) compared to others: MU 1 was relatively evenly represented by all locations (MR = 21, ER = 26, LHI = 24, LHIL = 29), but the remaining four MU (2, 3, 4 and 5) differed in representation of individuals from specific locations - MU 2 was LHI dominated (MR = 27, ER = 7, **LHI = 52**, LHIL = 13); MU 3 was under-represented by ER individuals (MR = 31, **ER = 7**, LHI = 31, LHIL = 31); MU 4 was ER dominated (MR = 17, **ER = 50**, LHI = 25, LHIL = 8) and MU 5 was LHIL dominated (MR = 18, ER = 12, LHI = 29, **LHIL = 41**). This indicates that three of the MU (2, 4 and 5) are overrepresented by three specific locations − LHI, ER and LHIL, respectively. In contrast, MR individuals were relatively evenly distributed across all five MU.

#### Population genetic analyses of mtDNA

based on an AMOVA, revealed two regional partitions (ER and MR vs LHI and LHIL) and all of the genetic variation (101.74%) was within locations, Φ_st_ = −0.017 (*p* = 0.8, Table S2), however, this was not significant. Pairwise F_st_ comparisons subsequently revealed no mtDNA genetic differentiation between locations (MR, ER, LHI, LHIL; F_st_ = −0.0029 to −0.008, *p* = 0.513 to 0.973, Table S4) and is consistent with the phylogenetic results.

#### Quantifying the level of evolutionary gene flow

**Figure 3:**
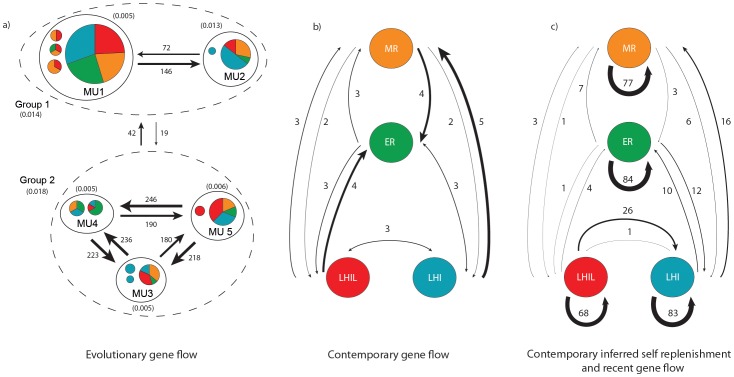
Migration rates among *Amphiprion mccullochi* locations. The thickness of the line is directly proportional to the number of migrants (M) and the colour of lines indicate predominant direction of gene flow. Population size (θ, within parentheses) is also shown for each location. a) Migrate-n evolutionary gene flow (mtDNA), b) Migrate-n contemporary gene flow (msatDNA) and c) BAYESASS analysis of self-replenishment (msatDNA) and recent migration shown as a percentage.

Bayesian analysis, informed by the phylogenetic structure, was performed using MIGRATE-n, because analyses based on spatial structure failed. High levels of evolutionary gene flow were indicated within - but less between groups: between Groups (i.e. Group 1 - Group 2) M ranged from 19 to 42 (Figure 3a). These values were 2- to 6- fold lower than evolutionary gene flow within groups: Group 1 (MU 1−2) M ranged from 72 to 146 (Figure 3a) and Group 2 (MU 3, 4, 5) M ranged from 180 to 246 (Figure 3a).

### Gene Flow between Locations - Contemporary Time Scales

#### Synopsis

msatDNA allele frequencies, genotypic distributions in space, genotypic assignments and genotypic posterior probability distributions suggested significant spatial partitions between *A. mccullochi* from the four locations in the latter three of the four analyses. Low levels of contemporary gene flow were detected between the four locations, consistent with the patterns of contemporary gene flow and with the high levels of inferred self-replenishment evident at all four locations (next section). This is in stark contrast to the patterns and levels of evolutionary gene flow.

#### Population genetic analyses of msatDNA

**Figure 4:**
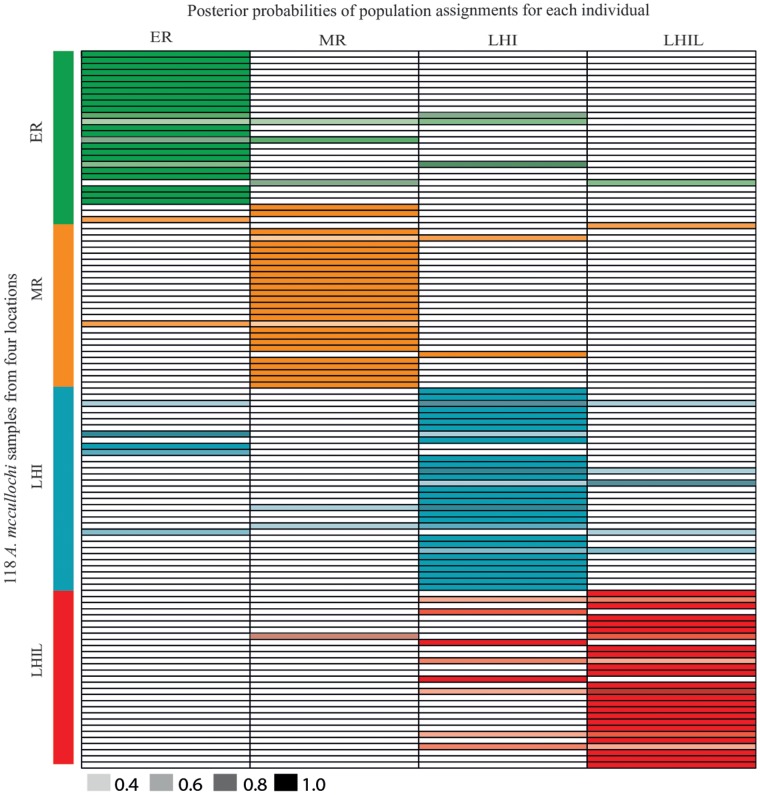
Posterior probability of assignment of each individual genotype to four *Amphiprion mccullochi* populations as indicated by DAPC. The names of the possible assignment populations are given on the x-axis. 118 genotypes are listed on the y-axis, along with the population from which they were sampled. Coloured bars corresponds to a 0.2 to 0.8 probability of assignment to a given population.

The statistically rigorous AMOVA found significant structure in the locus by locus msatDNA (Φ_st_ = −0.49 to 0.056, *p*<0.05, Table S3) and in the global AMOVA as a weighted average over all microsatellite loci (Φ_st_ = 0.007, *p* = 0.015, Table S2), with 99.34% of the genetic variation existing within locations. Raw msatDNA pairwise F_st_ comparisons also identified significant genetic partitioning between all locations (F_st_ = −0.004 to 0.026, *p* = 0.01 to 0.03, Table S4), but ENA corrected pairwise F_st_ values showed significant differentiation only between two of the four locations, ER and LHI (F_st_ = 0.014, *p*<0.05, Table S4). Discriminant analysis of principal components (DAPC) partitioned *A. mccullochi* into four spatially structured populations (Figure 2c). Using the four locations as *a priori* population criteria, DAPC assigned 76 to 80% of all individuals to the location from which they were sampled (assignment per population: 76% each for ER and LHI; 80% each for MR and LHI, Figure 4). The 95% genotypic inertia ellipses (GIE) for ER and LHIL did not overlap, whilst the 95% GIE for MR overlapped with all 95% GIEs from the remaining three locations. This is consistent with some ENA corrected pairwise F_st_ values and importantly, with the composition of MU 2, 4 and 5. Geographical structure in msatDNA data was also confirmed by GeneClass2 analyses, where only 5 individuals grouped with a location from which they were not sampled (MR = 1, ER = 1, LHIL = 3). Similarly, four geographically partitioned populations were identified by STRUCTURE analyses, as the likelihood of the marginal posterior probability distribution was highest when *K = *4.

#### Quantifying the level of contemporary gene flow

Contemporary gene flow between locations was a few orders of magnitude lower than evolutionary gene flow between locations using Migrate-n, with M values ranging from 2 to 5 (Figure 3b). This suggests that populations at each location are unlikely to be sustained from distant locations in the short term.

### Inferred Levels of Self-replenishment and Recent Migration

Despite weak genetic differentiation (F_st_) between locations, both DAPC and STRUCTURE partitioned the data into 4 distinct clusters. Used together, these programs are likely to be better than F_st_ values [60] at determining the appropriateness of a dataset for BAYESASS. Demographic independence is suggested for all location pairs except: LHIL to LHI (*m* = 26%), LHI to/from ER (*m* = 10 and 12%, respectively) and MR to LHI (*m* = 16%; Figure 3c). Conversely, high levels of self-replenishment (68 to 84%) were inferred at all four locations (Figure 3c). This indicates that each location is predominantly sustained by self-replenishment in the short term, rather than replenishment from distant locations.

### Population Genetic Diversities

*Amphiprion mccullochi* from all four locations had high haplotype diversity (*h*), nucleotide diversity (%π) and genotypic diversity (*gd*): *h* = 0.846 to 0.939,%π = 5.03 to 7.16, *gd* = 0.690 to 0.736 (Figure 2c). Total haplotype, nucleotide and genotypic diversities were also high, *h* = 0.897,%π = 5.70 and *gd* = 0.688 (Table S2) for this species. This is high genetic diversity and is unexpected for a low abundance endemic species, but is consistent with increased genetic diversity expected within locations when there is evolutionary connectivity between them (i.e. within location − high genetic diversity; between locations − low genetic diversity).

## Discussion

Isolated islands are global hotspots of endemicity for a range of coral reef organisms [64], [65] and determining the level and direction of gene flow [66] between locations is a fundamental step in establishing MPA networks that effectively conserve unique marine biodiversity. In this study, *A. mccullochi* was found to have: (i) sufficient gene flow between locations resulting in a lack of geographic partitioning over evolutionary time scales; (ii) genetically differentiated populations at all four sampled locations, due to low levels of contemporary gene flow between locations, despite the evolutionary homogenisation; (iii) demographic dependence between LHI and LHIL, LHI and ER and MR and LHI,yet high levels of inferred self-replenishment at all four locations and; (iv) high genetic diversity at all locations, despite high levels of inferred self replenishment. This is consistent with inter-location gene flow at evolutionary time scales.

### Gene Flow between Locations - Evolutionary Time Scales

The identification of discrete phylogenetic lineages or management units (MU) is critical for developing effective management strategies [67]. MU represent populations which rely on self regulation rather than immigration from external sources. Two distinct lineages with a total of 5 MU were suggested for *A. mccullochi* mtDNA. Despite this, the relative percentage of each location within MU suggests geographic structure. The occurrence of two lineages within a species has also been found for coral reef fishes on the Great Barrier Reef (GBR). Both *Plectropomus maculatus* and *Lutjanus carponotatus* show a lack of geographic partitioning along the GBR, yet display two distinct lineages, suggesting admixtures of differentiated lineages rather than stable populations [25]. A lack of geographical structure has also been found in endemic Hawaiian species *Chaetodon multicinctus*, *Chaetodon miliaris*, *Chaetodon fremblii* [68] and *Halichoeres ornatissimus* [69] and in numerous other widespread coral reef fish species including *S. frenatus* [70], *C. sordidus* [71], *Lethrinus miniatus* [72], *Pseudochromis fuscus* [73] and *Plectropomus leopardus* [74].

*A. mccullochi* showed high evolutionary gene flow between MU within lineages and to a far lesser extent, between lineages. Higher gene flow from Group 2 into Group 1 is clear, suggesting introgression of mtDNA (shown to be a result of historical hybridisation between *A. mccullochi* and its widespread sister species *A. akindynos*) [51]. In a similar way, the levels of evolutionary gene flow between three sympatric species pairs of three-spined stickleback (*Gasterosteus aculeatu*) have revealed natural hybridisation and break down of a species pair into a hybrid swarm [75]. In addition, evolutionary gene flow between locations has also been found in Red Sea reef fishes *Larabicus quadrilineatus, Chromis viridis* and *Pseudanthias squamipinnis* [76], [77]. Consequently, the lack of geographical structuring and observed spatial genetic homogeneity identified in this study of the endemic *A. mccullochi*, is likely due to high levels of evolutionary gene flow, which is sufficient for all locations to be connected on evolutionary time scales, thereby maintaining genetic homogeneity.

### Gene Flow between Locations - Contemporary Time Scales

*A. mccullochi* showed strong contemporary genetic differentiation between locations, consistent with other coral reef fish such as the Hawaiian endemic surgeonfish *Ctenochaetus strigosus* [78]. Strong discrepancies between evolutionary and contemporary levels of gene flow in *A. mccullochi* are a direct result of different spatial and temporal time scales. Discrepancies in gene flow, between time scales, has also been shown for *Lutjanus synagris* [79], *Plectropomus maculatus* and *Lutjanus carponotatus* [8], [9].

As previously highlighted, only a few individuals are needed over evolutionary time scales to ensure homogeneity across a species entire geographical range [28], [29]. However, models predict that this level of gene flow is not sufficient to sustain local populations and as a consequence, local populations must sustain themselves via self-recruitment or self-replenishment [40], [80]. Thus, although evolutionary gene flow is important, it is the dispersal rate of individuals that is of immediate interest to sustaining populations [81]. *A. mccullochi* showed very low levels of gene flow at contemporary timescales which is consistent with model prediction. The low levels of contemporary gene flow in this system most likely result from the short pelagic larval duration of *A. mccullochi* and the geographical isolation between locations enhanced by predominant east to west oceanographic currents limiting north-south gene flow between locations [53].

### Inferred Levels of Self-replenishment and Recent Migration

Demographic independence results from gene flow between two locations falling below 10% [82]. Thus, the high abundance of the McCulloch's anemonefish residing within the LHI lagoon will not directly sustain other locations in the short term, except outside the lagoon at LHI. Rather LHIL will help replenish LHI, which in turn will replenish ER, whilst both ER and MR will replenish LHI. This complex network of gene flow highlights the need to protect each location under one management strategy. Interestingly, the levels of inferred self-replenishment found in this study (≥68%) are remarkably similar to the estimated levels of self-recruitment in other congeneric anemonefish studies in Papua New Guinea (PNG) [83], [84]. These levels are also similar to those found in other reef fishes inhabiting islands including butterflyfish in PNG [85] and wrasse in the Caribbean [86], whose estimates of self-recruitment ranged from 30 to 60%. Possibly, the higher self-replenishment in *A. mccullochi*, compared to the above studies, results from the complete sampling of all locations leaving no 'ghost ' populations un-sampled. However, further investigation using direct methods (e.g. by using natural or artificial otolith tags of newly recruited juveniles [83], [84]) is necessary to validate the inferred levels of self-replenishment in *A. mccullochi*. This approach may not be appropriate for endemic species with low abundance. Given the rarity of *A. mccullochi* at MR and ER, parentage studies involving otolith tagging and the sacrificing of a high proportion of individuals may lead to local extinction at these sites.

### Population Genetic Diversities

*A. mccullochi* showed high genetic diversities despite its low abundance and high levels of inferred self replenishment. Similarly high genetic diversities have also been found in other coral reef fish including *Plectropomus maculatus*, *Lutjanus carponotatus* [25], *Lethrinus miniatus* [87] and damselfish on the Great Barrier Reef [71]. In *A. mccullochi* this higher than expected genetic diversity is most likely driven by bi-directional hybridisation with its sister species *A. akindynos* [51], a process which has also been documented in *Plectropomus leopardus* [74]. While high genetic diversities may provide some level of population resilience to environmental change, high levels of inferred self-replenishment make populations more vulnerable to extirpation due to low levels of replenishment from elsewhere via contemporary gene flow. Additionally, a cautious approach is required to prevent population losses, even those with high genetic diversity [88], as quantitative trait loci under selection at the peripheral edge of a species distribution range might have no genetic diversity remaining, despite neutral markers having relatively high genetic diversity in the same population [89]. Therefore, low levels of contemporary gene flow, coupled with high levels of self-replenishment have implications for the management, persistence and effective conservation of this endemic coral reef fish species – even if genetic diversity is high.

#### Threats and concerns

Conserving endemic species such as *A. mccullochi* presents a unique challenge to management. Although remote islands are largely unaffected by the pressures experienced by coastal reefs, a variety of anthropogenic threats still exist. These include sewage leaks and anemone bleaching due to increased temperatures [90]. The occurrence of these events at locations such as LHI lagoon [91] is a serious cause for concern [92] since 75% of *A. mccullochi* surveyed in 2009 resided in designated high-protection ‘sanctuary zones’ within the lagoon [48]. It follows then that protecting critical habitat (i.e. *Entacmaea quadricolor* anemones) and keeping the natural genetically distinct sub-populations (MU) of endemic fish intact, should be a priority of management plans. In addition, isolated locations that are predominantly dependent on self-recruitment are unlikely to be sustained by long distance transport over hundreds of kilometres [40], [80] and therefore unlikely to recover fast [93], [94]. Lastly, small, isolated populations are subject to genetic deterioration and, if habitat fragmentation increases in the future (due to habitat loss from climate change), gene flow may be further restricted, leading to inbreeding and an increase in extinction risk with as much as 29% reduced persistence times [95].

Climate change offers an additional suite of threats and concerns**.** LHI, like other isolated islands, is facing an escalation of threats (e.g. increasing intensity and frequency of cyclones, rising sea surface temperatures, ocean acidification) [50], with negative effects on biodiversity expected within the region. In the case of the McCulloch’s anemonefish and Hobbs et al. [48] noted in their surveys of LHI coral reefs that some of the host anemones were bleached (typically a response to elevated sea temperatures) [96]. As sea temperatures continue to increase due to global warming, the intensity and frequency of bleaching events is likely to increase, directly threatening the persistence of this obligate habitat specialist and potentially other coral reef fish. High genetic diversity is unlikely to overcome the loss of habitat in the time frames expected, particularly if the quantitative trait associated with specialised host use already has limited or no genetic diversity. With the expected increase in strength of the EAC bringing warmer waters to subtropical regions [97], these isolated island populations may at further risk of extinction if they can not tolerate elevated temperatures or extend their current geographic ranges.

#### Conclusion

The present study highlights the importance of estimating both evolutionary and contemporary levels of gene flow (connectivity) due to the different spatial and temporal scales at which these processes operate. While populations are primarily being maintained by self replenishment, exchange among islands over evolutionary time is critical to understanding patterns of genetic diversity and differentiation. Locations with high levels of self-replenishment (e.g. MR, ER, LHI) each require protection as they receive few dispersing larvae from each other. Locations with lower levels of self-replenishment (e.g. LHIL) are just as important to protect as they provide a dual benefit because they are a source for their own and other populations, aiding in rescue effects of depleted/extinct populations and enhancing genetic diversity. Thus both predominantly self-replenishing and predominantly dispersing locations should ideally be protected, from activities such as aquarium collecting, to maximise biodiversity conservation in low abundance endemics living on isolated reefs and islands. Although this study focused on a single coral reef species at four locations in the South-West Pacific Ocean, the region harbours 16 other species of endemic marine fishes, as well as numerous other endemic marine species that have similar geographic distributions as our study species. Thus patterns of gene flow and self-replenishment in *A. mccullochi* may be representative of other endemic species.

## Supporting Information

Table S1 **Summary statistics for 17 microsatellite loci Am1–24.**
(DOC)

Table S2 **AMOVA analysis for a) mtDNA sequences from** ***Amphiprion mccullochi*** **structured into geographic regions and b) global AMOVA weighted across all seventeen microsatellite loci.**
(DOC)

Table S3 **AMOVA fixation indices (Φ_st_) for** ***Amphiprion mccullochi*** **across all populations surveyed.**
(DOC)

Table S4 **Pairwise population Fst values for four populations of** ***Amphiprion mccullochi*** **using both d loop (mtDNA) and microsatellite (msat).** Pairwise population structures (F_st_) for four populations of *A. mccullochi*, using both d loop (mtDNA) and microsatellite (msat) loci showing raw and corrected Fst for null allele frequencies.
(DOC)

Table S5 **Sample sizes for D loop (total** ***n*** **= 105).**
(DOC)
