# Supplementary material for: Genetic Connectivity among and Self-Replenishment within Island Populations of a Restricted Range Subtropical Reef Fish
Source: PLoS One. 2012 Nov 21;7(11):e49660. doi: 10.1371/journal.pone.0049660 (PMC3504158; doi:10.1371/journal.pone.0049660)
Supplement: Table S1 — Summary statistics for 17 microsatellite loci Am1–24. (DOC) [file pone.0049660.s001.doc]

Table S1. Summary statistics for 17 microsatellite loci Am1-24. Sample sizes (N), observed number of alleles (Na), observed number of private alleles (Pa), observed heterozygosity (Ho), expected heterozygosity (He), the average inbreeding coefficient (F_IS_), probability of departure from HWE (pHWE) and significance of departure after FDR correction FDR (pFDR) for each locus at each population (significance of departure in bold, p < 0.05).

| Population | Am1 | Am4 | Am5 | Am6 | Am7 | Am9 | Am10 | Am11 | Am12 | Am14 | Am15 | Am17 | Am18 | Am19 | Am21 | Am22 | Am24 |
| --- | --- | --- | --- | --- | --- | --- | --- | --- | --- | --- | --- | --- | --- | --- | --- | --- | --- |
| **All (118)** |  |  |  |  |  |  |  |  |  |  |  |  |  |  |  |  |  |
| N | 115 | 115 | 113 | 114 | 111 | 114 | 115 | 115 | 110 | 109 | 91 | 95 | 103 | 83 | 85 | 93 | 110 |
| Na | 5 | 11 | 16 | 5 | 12 | 16 | 10 | 13 | 11 | 11 | 17 | 16 | 13 | 16 | 10 | 16 | 5 |
| Pa | 115 | 115 | 113 | 114 | 111 | 114 | 115 | 115 | 110 | 109 | 91 | 95 | 103 | 83 | 85 | 93 | 110 |
| Ho | 0.626 | 0.713 | 0.832 | 0.702 | 0.649 | 0.974 | 0.696 | 0.574 | 0.855 | 0.771 | 0.868 | 0.684 | 0.845 | 0.723 | 0.824 | 0.871 | 0.591 |
| He | 0.547 | 0.789 | 0.850 | 0.622 | 0.793 | 0.904 | 0.683 | 0.791 | 0.813 | 0.803 | 0.848 | 0.824 | 0.789 | 0.847 | 0.812 | 0.857 | 0.567 |
| Fis | -0.157 | 0.098 | 0.020 | -0.123 | 0.190 | -0.073 | -0.018 | 0.274 | -0.054 | 0.036 | -0.022 | 0.158 | -0.073 | 0.144 | -0.001 | -0.015 | -0.040 |
| pHWE | **0.013** | 0.367 | 0.998 | 0.646 | 0.130 | 0.789 | 0.999 | **0.000** | 0.647 | **0.000** | 1.000 | **0.000** | **0.000** | **0.000** | 0.433 | **0.000** | 0.400 |
| pFDR | 0.069 | 0.671 | 0.817 | 0.817 | 0.361 | 0.817 | 0.817 | **0.000** | 0.817 | **0.000** | 0.817 | **0.000** | **0.000** | **0.000** | 0.734 | **0.000** | 0.695 |
|  |  |  |  |  |  |  |  |  |  |  |  |  |  |  |  |  |  |
| **ER (25)** |  |  |  |  |  |  |  |  |  |  |  |  |  |  |  |  |  |
| N | 25 | 25 | 25 | 25 | 22 | 25 | 25 | 25 | 22 | 25 | 20 | 15 | 15 | 20 | 21 | 13 | 21 |
| Na | 4 | 10 | 10 | 4 | 6 | 13 | 5 | 9 | 8 | 9 | 13 | 10 | 7 | 11 | 8 | 13 | 4 |
| Pa | 0 | 0 | 2 | 0 | 0 | 0 | 0 | 1 | 0 | 0 | 2 | 2 | 2 | 2 | 0 | 2 | 0 |
| Ho | 0.800 | 0.640 | 0.840 | 0.680 | 0.500 | 0.880 | 0.720 | 0.560 | 0.909 | 0.880 | 0.850 | 0.800 | 0.867 | 0.600 | 0.905 | 0.846 | 0.524 |
| He | 0.554 | 0.774 | 0.868 | 0.566 | 0.729 | 0.912 | 0.756 | 0.805 | 0.789 | 0.806 | 0.845 | 0.784 | 0.767 | 0.824 | 0.804 | 0.891 | 0.490 |
| Fis | -0.426 | 0.192 | 0.053 | -0.181 | 0.335 | 0.055 | 0.068 | 0.323 | -0.129 | -0.071 | 0.020 | 0.015 | -0.096 | 0.295 | -0.101 | 0.090 | -0.045 |
| pHWE | 0.263 | **0.025** | 0.825 | 0.744 | **0.042** | 0.698 | 0.095 | **0.000** | 0.777 | 0.988 | 0.996 | 0.572 | **0.009** | **0.013** | 0.116 | 0.543 | 0.979 |
| pFDR | 0.546 | 0.109 | 0.817 | 0.817 | 0.162 | 0.817 | 0.314 | **0.000** | 0.817 | 0.817 | 0.817 | 0.817 | 0.057 | 0.069 | 0.361 | 0.817 | 0.817 |
|  |  |  |  |  |  |  |  |  |  |  |  |  |  |  |  |  |  |

**Table S3** *(Continued)*

| Population | Am1 | Am4 | Am5 | Am6 | Am7 | Am9 | Am10 | Am11 | Am12 | Am14 | Am15 | Am17 | Am18 | Am19 | Am21 | Am22 | Am24 |
| --- | --- | --- | --- | --- | --- | --- | --- | --- | --- | --- | --- | --- | --- | --- | --- | --- | --- |
| **MR (30)** |  |  |  |  |  |  |  |  |  |  |  |  |  |  |  |  |  |
| N | 33 | 31 | 29 | 33 | 32 | 33 | 33 | 33 | 32 | 28 | 23 | 28 | 31 | 23 | 24 | 28 | 32 |
| Na | 4 | 9 | 12 | 4 | 12 | 13 | 8 | 8 | 10 | 10 | 11 | 11 | 9 | 10 | 9 | 11 | 4 |
| Pa | 0 | 0 | 2 | 0 | 2 | 0 | 1 | 1 | 0 | 1 | 1 | 3 | 1 | 0 | 0 | 0 | 0 |
| Ho | 0.576 | 0.548 | 0.828 | 0.758 | 0.594 | 1.000 | 0.758 | 0.545 | 0.875 | 0.857 | 0.870 | 0.786 | 0.839 | 0.826 | 0.875 | 0.821 | 0.469 |
| He | 0.528 | 0.757 | 0.826 | 0.670 | 0.792 | 0.905 | 0.690 | 0.779 | 0.833 | 0.849 | 0.830 | 0.824 | 0.779 | 0.847 | 0.824 | 0.853 | 0.572 |
| Fis | -0.076 | 0.291 | 0.015 | -0.116 | 0.265 | -0.090 | -0.083 | 0.314 | -0.035 | 0.009 | -0.026 | 0.065 | -0.061 | 0.047 | -0.041 | 0.055 | 0.196 |
| pHWE | 0.876 | **0.018** | 0.670 | 0.460 | 0.273 | **0.018** | 0.998 | 0.154 | 0.910 | 0.698 | 0.998 | 0.270 | 0.937 | 0.784 | 0.627 | 0.704 | 0.595 |
| pFDR | 0.817 | 0.083 | 0.817 | 0.743 | 0.546 | 0.083 | 0.817 | 0.396 | 0.817 | 0.817 | 0.817 | 0.546 | 0.817 | 0.817 | 0.817 | 0.817 | 0.817 |
|  |  |  |  |  |  |  |  |  |  |  |  |  |  |  |  |  |  |
| **LHI (33)** |  |  |  |  |  |  |  |  |  |  |  |  |  |  |  |  |  |
| N | 33 | 31 | 29 | 33 | 32 | 33 | 33 | 33 | 32 | 28 | 23 | 28 | 31 | 23 | 24 | 28 | 32 |
| Na | 4 | 9 | 12 | 4 | 12 | 13 | 8 | 8 | 10 | 10 | 11 | 11 | 9 | 10 | 9 | 11 | 4 |
| Pa | 0 | 0 | 2 | 0 | 2 | 0 | 1 | 1 | 0 | 1 | 1 | 3 | 1 | 0 | 0 | 0 | 0 |
| Ho | 0.576 | 0.548 | 0.828 | 0.758 | 0.594 | 1.000 | 0.758 | 0.545 | 0.875 | 0.857 | 0.870 | 0.786 | 0.839 | 0.826 | 0.875 | 0.821 | 0.469 |
| He | 0.528 | 0.757 | 0.826 | 0.670 | 0.792 | 0.905 | 0.690 | 0.779 | 0.833 | 0.849 | 0.830 | 0.824 | 0.779 | 0.847 | 0.824 | 0.853 | 0.572 |
| Fis | -0.076 | 0.291 | 0.015 | -0.116 | 0.265 | -0.090 | -0.083 | 0.314 | -0.035 | 0.009 | -0.026 | 0.065 | -0.061 | 0.047 | -0.041 | 0.055 | 0.196 |
| pHWE | 0.876 | 0.018 | 0.670 | 0.460 | 0.273 | **0.018** | 0.998 | 0.154 | 0.910 | 0.698 | 0.998 | 0.270 | 0.937 | 0.784 | 0.627 | 0.704 | 0.595 |
| pFDR | 0.817 | 0.083 | 0.817 | 0.743 | 0.546 | 0.083 | 0.817 | 0.396 | 0.817 | 0.817 | 0.817 | 0.546 | 0.817 | 0.817 | 0.817 | 0.817 | 0.817 |
|  |  |  |  |  |  |  |  |  |  |  |  |  |  |  |  |  |  |

**Table S3** *(Continued)*

| Population | Am1 | Am4 | Am5 | Am6 | Am7 | Am9 | Am10 | Am11 | Am12 | Am14 | Am15 | Am17 | Am18 | Am19 | Am21 | Am22 | Am24 |
| --- | --- | --- | --- | --- | --- | --- | --- | --- | --- | --- | --- | --- | --- | --- | --- | --- | --- |
| **LHIL (30)** |  |  |  |  |  |  |  |  |  |  |  |  |  |  |  |  |  |
| N | 27 | 30 | 30 | 27 | 28 | 27 | 30 | 29 | 28 | 28 | 27 | 27 | 30 | 27 | 27 | 27 | 28 |
| Na | 3 | 11 | 9 | 4 | 10 | 13 | 5 | 7 | 8 | 8 | 11 | 9 | 9 | 13 | 9 | 10 | 4 |
| Pa | 0 | 1 | 0 | 0 | 0 | 1 | 1 | 1 | 0 | 0 | 2 | 1 | 0 | 4 | 0 | 1 | 0 |
| Ho | 0.556 | 0.900 | 0.800 | 0.667 | 0.786 | 1.000 | 0.600 | 0.621 | 0.821 | 0.750 | 0.889 | 0.593 | 0.867 | 0.704 | 0.778 | 0.889 | 0.571 |
| He | 0.543 | 0.819 | 0.844 | 0.620 | 0.851 | 0.907 | 0.635 | 0.782 | 0.833 | 0.763 | 0.875 | 0.823 | 0.812 | 0.853 | 0.800 | 0.843 | 0.554 |
| Fis | -0.005 | -0.082 | 0.069 | -0.056 | 0.095 | -0.084 | 0.072 | 0.223 | 0.032 | 0.035 | 0.003 | 0.297 | -0.050 | 0.193 | 0.046 | -0.036 | -0.013 |
| pHWE | **0.007** | 0.901 | 0.910 | 0.724 | 0.972 | 0.203 | 0.981 | 0.127 | 0.830 | 0.898 | 0.647 | 0.219 | **0.034** | 0.346 | 0.446 | 0.502 | 0.192 |
| pFDR | **0.049** | 0.817 | 0.817 | 0.817 | 0.817 | 0.470 | 0.817 | 0.361 | 0.817 | 0.817 | 0.817 | 0.491 | 0.139 | 0.650 | 0.738 | 0.793 | 0.460 |
