# Supplementary material for: Genetic Connectivity among and Self-Replenishment within Island Populations of a Restricted Range Subtropical Reef Fish
Source: PLoS One. 2012 Nov 21;7(11):e49660. doi: 10.1371/journal.pone.0049660 (PMC3504158; doi:10.1371/journal.pone.0049660)
Supplement: Table S2 — AMOVA analysis for a) mtDNA sequences from Amphiprion mccullochi structured into geographic regions and b) global AMOVA weighted across all seventeen microsatellite loci. (DOC) [file pone.0049660.s002.doc]

Table S2: AMOVA analysis for a) mtDNA sequences from *Amphiprion mccullochi*

structured into geographic regions and b) global AMOVA weighted across all seventeen microsatellite loci.

Significant *p*-values are in bold.

| **Source of variation** | **Variance component** | **Percentage of variation** | **F-statistics fixation indices**  **(p-value)** |
| --- | --- | --- | --- |
| ***a) Region*** |  |  |  |
| Amoung groups | -0.02 | -0.17 | F_ct_ = -0.002  (0.688) |
| Amoung populations  within groups | -0.14 | -1.57 | F_sc_ = -0.016  (0.682) |
| Within populations | 9.10 | 101.74 | F_st_ = -0.017  (0.800) |
| ***b) Microsatellite*** |  |  |  |
| Amoung groups | 0.03 | 0.41 | F_ct_ = 0.004  (0.037) |
| Amoung populations  within groups | 0.02 | 0.26 | F_sc_ = 0.003  (0.235) |
| Within populations | 6.70 | 99.34 | F_st_ = 0.007  **(0.015)** |
