# Supplementary material for: Genetic Connectivity among and Self-Replenishment within Island Populations of a Restricted Range Subtropical Reef Fish
Source: PLoS One. 2012 Nov 21;7(11):e49660. doi: 10.1371/journal.pone.0049660 (PMC3504158; doi:10.1371/journal.pone.0049660)
Supplement: Table S3 — AMOVA fixation indices (Φst) for Amphiprion mccullochi across all populations surveyed. (DOC) [file pone.0049660.s003.doc]

Table S3: AMOVA fixation indices (Φ_st_) for *Amphiprion mccullochi* across all populations surveyed

|  | Marker class and analysis | | | | | |
| --- | --- | --- | --- | --- | --- | --- |
|  | | Raw msat | Msat corrected for  null allele freq. | | Standardised msat | |
| Average | **0.007** | | | 0.005 | | 0.019 |
| Am1 | -0.004 | | | **-0.001** | | **-0.049** |
| Am4 | -0.004 | | | **-0.007** | | **0.032** |
| Am5 | 0.008 | | | 0.007 | | 0.076 |
| Am6 | 0.007 | | | 0.009 | | **0.048** |
| Am7 | 0.001 | | | **-0.005** | | **0.021** |
| Am9 | -0.003 | | | **-0.002** | | **0.053** |
| Am10 | 0.013 | | | **0.013** | | **0.043** |
| Am11 | 0.010 | | | 0.004 | | **0.025** |
| Am12 | 0.007 | | | 0.008 | | **-0.024** |
| Am14 | 0.011 | | | **0.010** | | **0.027** |
| Am15 | 0.006 | | | 0.006 | | **0.047** |
| Am17 | 0.010 | | | **0.011** | | **-0.016** |
| Am18 | 0.003 | | | 0.004 | | **-0.025** |
| Am19 | 0.002 | | | **0.001** | | **0.056** |
| Am21 | -0.008 | | | **-0.008** | | **0.041** |
| Am22 | 0.004 | | | 0.004 | | **-0.031** |
| Am24 | **0.031** | | | **0.031** | | **-0.002** |

Raw population differentiation from microsatellite allele frequencies for each individual loci and the average across all loci, population differentiation corrected for null allele frequencies using the ENA correction and standardized population differentiation for and across all loci (Φ_st_). All values in bold are significant to the p<0.05 (i.e. 95% confidence interval).
