# Supplementary material for: Genetic Connectivity among and Self-Replenishment within Island Populations of a Restricted Range Subtropical Reef Fish
Source: PLoS One. 2012 Nov 21;7(11):e49660. doi: 10.1371/journal.pone.0049660 (PMC3504158; doi:10.1371/journal.pone.0049660)
Supplement: Table S4 — Pairwise population Fst values for four populations of Amphiprion mccullochi using both d loop (mtDNA) and microsatellite (msat). Pairwise population structures (Fst) for four populations of A. mccullochi, using both d loop (mtDNA) and microsatellite (msat) loci showing raw and corrected Fst for null allele frequencies. (DOC) [file pone.0049660.s004.doc]

Table S4: Pairwise population Fst values for four populations of *Amphiprion mccullochi* using both d loop (mtDNA) and microsatellite (msat). Pairwise population structures (F_st_) for four populations of *A. mccullochi*, using both d loop (mtDNA) and microsatellite (msat) loci showing raw and corrected Fst for null allele frequencies.

|  | D-loop | | | |  | Raw msat | | | |  | Corrected msat | | | |
| --- | --- | --- | --- | --- | --- | --- | --- | --- | --- | --- | --- | --- | --- | --- |
|  | ER | MR | LHI-N | LHI-L |  | ER | MR | LHI-N | LHI-L |  | ER | MR | LHI-N | LHI-L |
| ER |  | 0.513 | 0.604 | 0.378 |  |  | **0.010** | **0.010** | **0.010** |  |  | >0.05 | >0.05 | **<0.05** |
| MR | -0.013 |  | 0.973 | 0.490 |  | 0.023 |  | **0.020** | **0.010** |  | 0.005 |  | >0.05 | >0.05 |
| LHI-N | -0.024 | -0.029 |  | 0.550 |  | 0.016 | 0.007 |  | **0.030** |  | 0.005 | 0.002 |  | >0.05 |
| LHI-L | -0.008 | -0.009 | -0.018 |  |  | 0.026 | 0.023 | 0.004 |  |  | 0.014 | 0.005 | 0.000 |  |

Fst values below diagonal. Significant p-vlaues are in bold (p > 0.05) above diagonal. Raw population differentiation from microsatellite allele frequencies and associated *p*-values. Corrected population differentiation for null allele frequencies at 95% CI (all *p*-values > 0.05) using the ENA correction. FDR correction of raw and corrected msat F_st_ *p*-values.
