# Supplementary material for: Genetic Connectivity among and Self-Replenishment within Island Populations of a Restricted Range Subtropical Reef Fish
Source: PLoS One. 2012 Nov 21;7(11):e49660. doi: 10.1371/journal.pone.0049660 (PMC3504158; doi:10.1371/journal.pone.0049660)
Supplement: Table S5 — Sample sizes for D loop (total n = 105). (DOC) [file pone.0049660.s005.doc]

Table S5: Sample sizes for D loop (total *n* = 105)

|  | *n*  (D loop) | *n_h_* | *h* | *π*(%) | *n* (msat) | *gd* | N_a_ | P_a_ | H_o_ | H_e_ | Fis |
| --- | --- | --- | --- | --- | --- | --- | --- | --- | --- | --- | --- |
| All | 105 | 31 | 0.897 | 5.70 | 118 | 0.688 | 8.7 | 50 | 0.752 | 0.773 | 0.02 |
| ER | 21 | 12 | 0.914 | 5.13 | 25 | 0.736 | 8.5 | 13 | 0.753 | 0.763 | 0.04 |
| MR | 22 | 13 | 0.939 | 5.03 | 30 | 0.691 | 8.9 | 12 | 0.750 | 0.781 | 0.06 |
| LHI-N | 31 | 13 | 0.882 | 7.16 | 33 | 0.704 | 9.1 | 12 | 0.754 | 0.774 | 0.04 |
| LHI-L | 26 | 11 | 0.846 | 5.11 | 30 | 0.690 | 8.4 | 12 | 0.752 | 0.774 | 0.05 |

Number of haplotypes (*n_h_*), haplotype diversity (*h*), nucleotide diversities (*π*) of D loop for all regions and populations of *Amphiprion mccullochi*. Sample sizes for msats (total n = 118), genetic diversity (*gd*) average number of alleles per locus (Na), observed number of private alleles (Pa), observed heterozygosity (H_o_) and expected heterozygosity (H_e_) and the inbreeding coefficient (Fis) averaged over seventeen microsatellite loci for four populations in three regions.
